# Diagnostic and Therapeutic Values of Angiogenic Factors in Endometrial Cancer

**DOI:** 10.3390/biom12010007

**Published:** 2021-12-21

**Authors:** Luka Roškar, Irena Roškar, Tea Lanišnik Rižner, Špela Smrkolj

**Affiliations:** 1 Department of Gynaecology and Obstetrics, Faculty of Medicine, University of Ljubljana, 1000 Ljubljana, Slovenia; luka.roskar@outlook.com; 2 Institute of Biochemistry, Faculty of Medicine, University of Ljubljana, 1000 Ljubljana, Slovenia; ena.roskar@hotmail.com (I.R.); tea.lanisnik-rizner@mf.uni-lj.si (T.L.R.); 3 Division of Gynaecology and Obstetrics, University Medical Centre, 1000 Ljubljana, Slovenia

**Keywords:** biomarkers, angiogenesis, endometrial cancer, angiogenic factors, anti-angiogenic therapy

## Abstract

Endometrial cancer (EC) is the most frequent gynecological malignancy in developed countries and requires a relatively invasive diagnostic evaluation and operative therapy as the primary therapeutic approach. Angiogenesis is one of the main processes needed for cancer growth and spread. The production of angiogenic factors (AFs) appears early in the process of carcinogenesis. The detection of AFs in plasma and tissue and a better understanding of the angiogenic properties of EC may contribute not only to earlier but also more specific diagnosis and consequently tailored and individual therapeutic approaches. AFs and their receptors also have high potential as binding sites for targeted cancer therapy. In this review, we discuss angiogenesis in EC and the characteristics of the AFs that most contribute to angiogenesis in EC. We also highlight therapeutic strategies that target angiogenesis as potential EC therapy.

## 1. Introduction

Endometrial cancer (EC) is the most frequent gynecological malignancy in the developed world, and its incidence strongly depends on several risk factors [1]. One of the most prominent risk factors is obesity [2,3]. Adipose tissue serves as a storage for hormones that promote the proliferation of endometrial tissue, which leads to malignant alterations. Once endometrial cells become malignant, they obtain the ability of uncontrolled fast growth, which requires a good supply of oxygen and nutrients. Such supply via diffusion is extremely limited and can only support tumors smaller than 1–2 mm in diameter [4]. For larger tumors, EC cells must start to produce angiogenic factors (AFs), i.e., special cytokines, which are secreted into the surrounding tissue and nearby vessels, causing angiogenesis and the delivery of nutrients to the cancerous cells. Elevated AF levels can also be detected in the systemic circulation, indicating a potential non-invasive strategy to detect cancer in its early stages [5]. 

Altered AF levels in plasma and tissue and understanding of angiogenesis in EC may contribute to not only earlier but also more specific diagnosis. Furthermore, AFs and their receptors are also potential binding sites for targeted cancer therapy and may contribute to more tailored therapeutic approaches to EC. In this review, we discuss angiogenesis in EC, the characteristics of the AFs that contribute most to angiogenesis in EC, and also inhibition of angiogenesis as a potential targeted therapy for EC.

## 2. Methodology

We searched PubMed and ClinicalTrials.gov for journal articles, including original articles, reviews, and meta-analyses, published before November 2021, that described research, mechanisms of action, and prospective clinical trial data regarding angiogenesis and anti-angiogenic treatment in EC and gynecological cancers. Duplicate hits were discarded. Unrelated studies were excluded through careful browsing of the title, abstract, and/or whole text of each publication. This review contains information and data from 119 papers, narrowed down from 201 relevant articles.

## 3. Endometrial Cancer

EC is a leading gynecological malignancy in the developed world and the sixth most common female cancer worldwide [6]. Increasing age and elevated estrogen levels are well-known risk factors for EC. Thus, the incidence of EC is rapidly increasing due to increased life expectancy and obesity, which causes elevated estrogen levels [7,8].

According to endocrine and metabolic disturbances, EC is classified into two types. Well- or moderately differentiated endometrioid EC represents prognostically favorable type 1 EC, whereas poorly differentiated endometrioid EC represents prognostically less favorable type 2 EC with a tendency for deep myometrial invasion and metastasis. The average 5-year overall survival (OS) rates are 85.6% and 58.8% for type 1 and 2 EC, respectively [9,10].

While this classification into two types is still widely in use, a newer molecular classification was proposed in 2013 by The Cancer Genome Atlas (TCGA) Research Network, exclusively based on a profound molecular characterization of the tumors. It divides EC into four categories: POLE ultramutated, microsatellite instability hypermutated, copy-number low, and copy-number high [11,12]. Recently, it has been demonstrated that integrating molecular profiling into a daily routine is possible, and this is expected to optimize treatment decisions [13]. However, currently, the diagnosis and treatment strategy for EC are still mostly decided based on histological findings of endometrial biopsies [14]. The most important histological findings, which determine the extension of surgical therapy, are the type and grade of EC along with the presence of deep myometrial or lymphovascular invasion [7,15]. In terms of histopathology, endometrioid EC is categorized into three grades: well-differentiated (G1), moderately differentiated (G2), and poorly differentiated (G3) [16].

## 4. Angiogenesis

The formation of new vessels from pre-existing vessels is termed angiogenesis, which occurs during normal healing after tissue trauma and is mainly triggered by tissue ischemia. Low cellular oxygen levels cause the production of pro-angiogenic factors, their secretion into the surrounding tissue, and binding to capillaries or arterioles, provoking the growth of new vessels. This physiological process is highly regulated by pro- and anti-angiogenic molecules, mostly cytokines, commonly named AFs. Angiogenesis is also important in the female reproductive system, in which it allows monthly menstrual cycles and successful pregnancy. Pathological angiogenesis occurs in certain diseases, e.g., endometriosis, diabetic retinopathy, rheumatoid arthritis, and cancer. As extensive laboratory and clinical data suggest, it is one of the most important processes in the pathogenesis of cancer, as it is required for tumor growth and metastatic spread to other organs. Insufficient inhibition of angiogenesis enables the development and fast growth of tumors [4,17,18].

**Figure 1:**
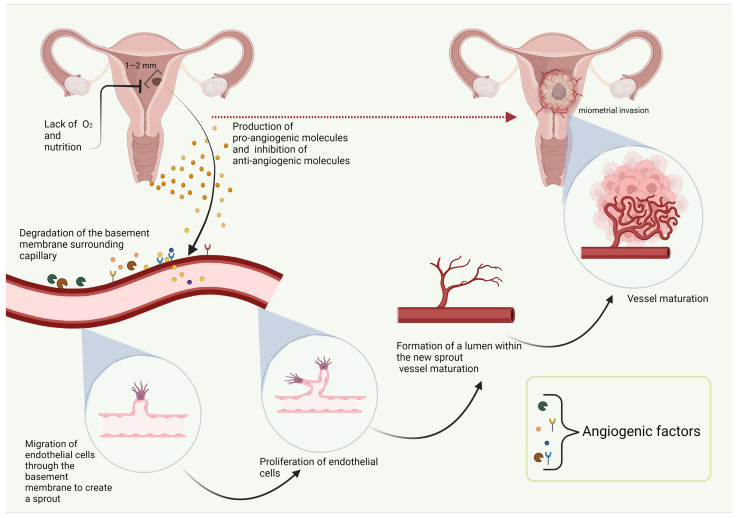
Angiogenesis in endometrial cancer. Created with BioRender.com.

Vessel growth occurs via four different mechanisms of angiogenesis: sprouting, intussusception, elongation, and incorporation of endothelial progenitor cells into new microvessels. Sprouting angiogenesis is the most efficient type of vascularization. Endothelial cells produce proteases, which break down the basement membrane and allow endothelial cells to migrate toward the circulating AFs. New microvessels are stabilized by pericytes and smooth muscle cells. Intussusceptive angiogenesis is characterized by the splitting of an existing blood vessel into two separate vessels. Endothelial cells migrate inward and create a network of new vessels. Elongation is the mechanism that most frequently occurs during organism growth [18,19]. The angiogenic processes during EC growth are shown in Figure 1. 

### 4.1. Angiogenesis in the Endometrium

The endometrium is, in comparison to other tissues, cyclically exposed to extensive hormonal changes. During each monthly cycle, ovarian hormones trigger angiogenic processes and regeneration of the endometrium, which is followed by subsequent blood vessel loss. Therefore, endometrial tissue produces both pro- and anti-AFs [18,20]. The expression of pro-AFs is the lowest during menstruation and increases during the early proliferative phase. Vascular growth starts in the proliferative phase and continues throughout the secretory phase of the menstrual cycle [21]. Vascular expansion in normal endometrium occurs by elongation and intussusceptive angiogenesis, which occurs rapidly (within minutes or hours) and does not rely on endothelial cell proliferation [10,11].

### 4.2. Angiogenesis in Cancer

During the initial stage of tumor growth and development (i.e., before the tumor’s size exceeds 1–2 mm^3^), the tumor is independent of the vascular network, as nutrients and oxygen can be obtained via diffusion. During the later stages of carcinogenesis, such nutrient supply becomes insufficient. Due to rapid tumor growth, high interstitial pressure, and larger distances between cancer cells and capillaries, hypoxia occurs in solid tumors. Hypoxia is an important controller of the angiogenic switch that is mainly regulated by hypoxia-inducible factor-1α (HIF-1α), a transcription factor that activates the transcription of a set of key genes involved in cell survival under hypoxic conditions, e.g., those involved in initiating angiogenesis [22]. In this way, new vasculature is formed in and around the tumor that provides essential nutrients and oxygen for the tumor cells and allows continuous growth and unlimited proliferation. Simultaneously, this vascular network is used for metastatic spread to other organs [4,23]. The density of the capillary network has been shown to be a prognostic factor for EC. Blood microvessel density was associated with deeper myometrial invasion, positive lymphovascular invasion, positive lymph node metastasis, and poor overall survival in EC patients [24].

### 4.3. Diagnostic Value of AFs

Normal angiogenesis is regulated by both molecular activators and inhibitors. In cancer, the balance between them is disturbed in favor of angiogenic activators. More than a few dozen different proteins have been identified as pro-AFs, including growth factors, cytokines, proteases, protease inhibitors, trace elements, oncogenes, and endogenous modulators [25]. The production of AFs by cancer cells alters AF levels in the surrounding tissue and blood plasma. Altered AF levels may thus represent potential markers that could detect cancer from blood plasma samples in the early and prognostically favorable stages of cancer [26]. AF plasma concentrations could also represent an important additional diagnostic tool for a more precise diagnosis of EC, which could also guide decision making regarding the extent of surgical treatment.

In the current review, we focus on the factors that seem to exhibit the highest potential for the diagnosis and prognosis of EC. We also highlight the existing and novel therapeutic strategies for targeting angiogenesis in EC.

## 5. Angiogenic Diagnostic and Therapeutic Biomarkers

### 5.1. Vascular Endothelial Growth Factor

Vascular endothelial growth factor (VEGF) is the most potent and specific endothelial cell growth factor. It plays a critical role in initiating physiological and pathological angiogenesis, lymphangiogenesis, and vasculogenesis [27,28]. VEGF is also secreted by adipose tissue and is thus directly related to obesity [29]. The VEGF family is composed of five ligands (VEGF-A, -B, -C, -D, and -E) and three receptors (VEGFR1, VEGFR2, and VEGFR3). The action of VEGF-A and -B through VEGFR1 is responsible for the formation of new vessels. VEGF-A, -B, -C, and -D also act through VGFR2, which activates angiogenesis, proliferation, and migration. The action of VEGF-C and VEGF-D through VEGFR3 results in lymphangiogenesis, proliferation, and migration [30,31]. VEGFs and VEGFRs are overexpressed under hypoxic conditions by HIF-1α and upregulated by leptin [32,33]. 

It is generally considered that the expression levels of AFs, among which VEGF is one of the most important, reflect the aggressiveness of the tumor [25]. VEGF overexpression in tumor cells enhances tumor growth and metastasis in several malignancies, including EC. Immunohistochemically, 63% of ECs express VEGF-A, 55% VEGFR2, and 26% VEGFR3. VEGFR3 was also significantly correlated with tumor stage, with a trend toward poorer disease-free survival [34]. Chen et al. evaluated 53 EC patients and found that overexpressed cytosolic VEGF, along with cancer histological grade, is an independent prognostic factor for disease-free survival [35]. This is supported by other studies that demonstrated that VEGF is associated with poor outcomes in EC patients and is an important marker for predicting disease-free 5-year survival rates in EC patients [36,37].

According to the results from a plethora of other studies by different authors, increased serum and EC tissue VEGF levels are significantly correlated with the clinical stage and histological grade of the tumor. Some studies have found that elevated VEGF expression is associated with deep myometrial invasion, poorly differentiated tumors, histological type, intratumoral microvessel density, and lymph node metastasis in EC patients [36,38,39,40,41,42]. By contrast, other studies found no correlation between VEGF expression and histological type or grade, depth of myometrial invasion, or lymphovascular invasion [37,43]. However, the latter two studies did indeed show that VEGF expression was higher in adenocarcinoma samples than that in normal endometrial tissue and that VEGF is an important indicator of poor prognosis.

### 5.2. Angiopoietins and Tie2

Angiopoietins are a family of growth factors that act as ligands for tyrosine kinase receptor Tie2, which is expressed on endothelial cells. Angiopoietin-1 (Ang-1) and angiopoietin-2 (Ang-2) share 60% of sequence homology and bind to Tie2 with equal affinity. While Ang-1 is pro-angiogenic and widely expressed in adult tissues, Ang-2 is only expressed at sites of vascular remodeling, acts as a functional antagonist of Ang-1, and leads to vessel regression in tumors in the absence of VEGF-A. Although Ang-2 reduces vascular integrity, it may make endothelial cells more responsive to proliferative VEGF signals [44,45,46,47]. 

Angiopoietins are thought to be useful diagnostic biomarkers of carcinogenesis. Ang-1 is moderately overexpressed by many tumor cells, and pronounced Tie2 expression can be detected throughout the tumor vasculature. Ang-2 transcription is upregulated in tumor-associated endothelium, and circulating Ang-2 levels have emerged as robust biomarkers of tumor progression for several different tumors [46].

According to Wang et al. [48], higher Ang-2 levels, together with higher VEGF-A levels, affect neo-angiogenesis as well as the instability of new blood vessels. A meta-analysis carried out by Xu et al. [49] showed that elevated Ang-2 levels in lung cancer are associated with poor prognosis. Furthermore, it has been proposed by various authors that Ang-2 is also a marker of obesity [50,51] and is possibly associated with the development of obesity-related diseases, such as EC. Holland et al. [52] demonstrated higher Ang-2 mRNA levels in EC than those in benign endometrium. Terlikowska et al. found that in EC type 1 patients, serum Ang-2 levels increased with the depth of myometrial infiltration, lymphovascular infiltration, and age and were highest in patients in FIGO stage III–IV. This suggests a key role of Ang-2 in tumor development and metastasis [53]. 

The soluble form of the Tie2 receptor (sTie2) results from proteolytic ectodomain shedding and has been detected in the circulatory system. Elevated sTie2 levels have been associated with certain neoplastic and non-neoplastic diseases and have been explored as circulating biomarkers [46]. However, in our previous study, we detected significantly lower sTie2 plasma levels in patients with the endometrioid type of EC compared to patients with benign gynecological pathologies [5]. 

Currently, the results from different studies regarding the use of angiopoietins as biomarkers for gynecological cancer are not conclusive. Saito et al. [43] detected higher VEGF and Tie2 expression and lower Ang-1 and Ang-2 expression in adenocarcinoma than that in normal epithelial cells. As a receptor for both Ang-1 and Ang-2, Tie2 has both pro- and anti-angiogenic properties [54]. More clinical studies on circulating angiopoietins and Tie2 are necessary to conclusively determine their use as diagnostic and prognostic biomarkers.

### 5.3. Granulocyte Colony-Stimulating Factor

Granulocyte colony-stimulating factor (G-CSF) is a cytokine most well-known for its role in the maturation and mobilization of bone marrow neutrophils. It has been used to treat chemotherapy-induced neutropenia for over 20 years [55]. By contrast, clinical reports have shown that it is also highly expressed in certain tumors in which it promotes malignant progression by facilitating tumor angiogenesis. G-CSF also promotes metastasis and is related to poor prognosis and decreased overall patient survival [56,57]. G-CSF exerts tumor-promoting effects on both the cells and microenvironment of tumors. Clinically, the diagnostic criteria for G-CSF-producing tumors are extreme leukocytosis and white blood cell count reduction after tumor resection. Immunohistochemically, positive cytoplasmic G-CSF staining is observed in tumor cells [58].

The aggressive nature of G-CSF-producing cancers was reported in several malignancies, including uterine and cervical carcinomas, which showed significantly elevated tumor-derived G-CSF levels and decreased overall survival [59]. Studies on uterine cervical cancer have also demonstrated that G-CSF produced by tumor cells causes tumor-related leukocytosis and that G-CSF-induced myeloid-derived suppressor cells are responsible for the rapidly growing and radio-/chemo-resistant nature of these tumors [60]. Tumor-related leukocytosis is observed in approximately 10–15% of patients with gynecological cancer and is associated with poor prognosis [59,61]. Myeloid-derived suppressor cells, a heterogeneous population of the myeloid lineage, induced by pro-inflammatory cytokine growth factors (including G-CSF) produce matrix metalloproteinase-9, which increases the bioavailability of VEGF, angiogenesis, tumor cell extravasation, and metastatic nodule formation in vivo [62,63].

In 2018, Sasano et al. investigated the metastatic potential of uterine cervical and endometrial cancer that displays tumor-related leukocytosis. They observed greater G-CSF immunoreactivity and serum levels in neutrophilia-positive patients with cervical and endometrial tumors. Their study showed that uterine cancer involving pretreatment tumor-related leukocytosis or neutrophilia is a distinct clinical entity with a highly metastatic nature [64]. In 2019, Yokoi et al. analyzed clinical data from 900 EC patients and showed that tumor-derived G-CSF and G-CSF-mediated IL-6 production from the tumor microenvironment are involved in the development of leukocytosis and thrombocytosis. They demonstrated that concurrent pretreatment leukocytosis and thrombocytosis is an independent poor prognostic factor in EC patients [65].

### 5.4. Leptin

Obesity is a well-known risk factor for the development of EC [66]. Among all obesity-related neoplasms, the incidence rate and mortality of EC are the most strongly associated with an increased BMI [66]. Adipose tissue is an endocrine organ, synthesizing so-called adipokines—biologically active substances participating in cell growth and differentiation, angiogenesis, apoptosis, and carcinogenesis. Adiponectin and leptin are the most important adipokines during EC development. According to many studies in recent decades, they can be used, independently or as a leptin/adiponectin ratio, as new markers in determining the potential risk of EC [67,68,69,70].

Leptin plays a key role as a hormone that regulates body mass homeostasis by regulating energy balance and glucose metabolism. During carcinogenesis, leptin promotes tumor angiogenesis, proliferation, migration, and invasion and suppresses apoptosis of cancer cells [67,71]. Additionally, large amounts of adipokines and other cytokines promote the recruitment of macrophages and impair the function of adipocytes, which ultimately induces chronic inflammation and contributes to the process of carcinogenesis [72]. Recent research has found higher leptin levels in EC patients, particularly in EC at higher stages and with lower grades of histopathological differentiation. Patients with higher leptin serum levels had an increased risk for lymph node metastases and lymphovascular invasion. Furthermore, the depth of myometrial invasion correlated with leptin levels, and positive leptin and leptin receptor status was shown to influence the survival rate of EC patients [73,74,75,76].

Boron et al. reported overexpression of leptin and its receptors in endometrioid EC both at the mRNA and protein levels [73]. Wang et al. performed a meta-analysis from which they concluded the following: 1) a high leptin level is an independent risk factor for EC and 2) contrary to other research [73,77,78], leptin levels in EC are independent of BMI [79]. This supports findings that leptin is further involved in endometrial carcinogenesis via other pathways, including the promotion of proliferation and angiogenic differentiation of endothelial cells, as was shown in vitro and in vivo [80,81,82].

Through its ability to promote angiogenesis, leptin plays a crucial role in carcinogenesis. It induces proliferation and angiogenic differentiation of endothelial cells, upregulates VEGF/VEGFR2, and transactivates VEGFR2 independently of VEGF [83]. It also induces two AFs: IL-1 and Notch that can increase VEGF expression [84]. Additionally, leptin induces the secretion and synthesis of proteases and adhesion molecules needed for angiogenesis [85]. It further affects stromal cells and tumor-associated macrophages, which express the leptin receptor and secrete VEGF and IL-1, respectively [86].

### 5.5. Other AFs

Neuropilin-1 and -2 are VEGF co-receptors. They mediate VEGF signaling but have been much less studied than VEGFR. An immunohistochemical study by Oplawski et al. [87] found that neuropilin-1 and -2 were not expressed in normal tissue. Neuropilin-1 was expressed in cancer cells and at distinctly higher levels in G2 and G3 compared to G1. Neuropilin-2 was expressed in malignant endothelium and correlated with tumor grade. Thus, the authors suggested that neuropilins may be candidates for complementary diagnostic molecular markers. Another study demonstrated that atypical neuropilin-1 expression in endometrial tissue may serve as a biomarker for metastatic endometrial tumors [88].

IL-8 is a pro-inflammatory cytokine expressed by macrophages and fibroblasts. It is a chemo-attractant and mediates angiogenesis [89], including tumor angiogenesis in many pathologies, e.g., EC [90]. A significant correlation was found between cancer staging and IL-8 expression [91]. Kotowicz et al. [92] demonstrated the clinical usefulness of IL-8 measurements as potential prognostic factors in type 1 EC, in which elevated pretreatment IL-8 serum levels were associated with shorter disease-free and overall survival. IL-8 is also produced and secreted by adipocytes, and its levels increase with BMI and waist circumference, which is also associated with EC incidence and outcomes [93].

## 6. Anti-Angiogenic Treatment of EC

The standard treatment of EC consists of surgery with or without adjuvant radiotherapy and/or chemotherapy, depending on the risk of disease recurrence [94]. EC at the advanced stage has a very poor prognosis under conventional treatment: the 5-year overall survival rate is 40–65% for stage III and 15–17% for stage IV of the disease [95]. Due to the lack of effective treatments, treating patients in an advanced-stage, recurrent, or refractory setting is very challenging. However, advances in our understanding of the molecular mechanisms of EC progression have revealed novel binding sites for targeted therapies, which are emerging as innovative and promising cancer treatment strategies. 

Various potential therapeutic approaches are being investigated. Due to the importance of new vasculature for tumor growth, efforts have been made to develop anti-angiogenic therapies for cancer treatment in recent decades. The main classes of anti-angiogenic agents are anti-VEGF monoclonal antibodies (e.g., bevacizumab), soluble VEGFRs (e.g., aflibercept), inhibitors of angiopoietin-Tie2 receptor (e.g., trebananib), and tyrosine kinase inhibitors (e.g., brivanib, cediranib, nintedanib, sunitinib, and lenvatinib) [96].

The earliest exploration of anti-angiogenic agents in EC was a phase-II study of thalidomide in refractory EC performed by the Gynecologic Oncology Group Foundation [97]. It demonstrated an association between elevated plasma VEGF levels and poor prognosis, while thalidomide exhibited limited ability to delay the progression or reduce angiogenic marker levels. Nonetheless, several approaches have been developed to block VEGF action and have achieved good clinical efficacy, including blocking antibodies, decoy receptors, and small interfering RNA against VEGF-A [98,99,100]. The best-known anti-angiogenic agent for gynecological malignancies is bevacizumab, a humanized anti-VEGF monoclonal antibody, currently approved by the FDA as a combination and/or maintenance treatment for certain ovarian and cervical cancer patients. Several phase-II studies have been conducted with bevacizumab in EC patients (Table 1); however, the results of phase-III studies in EC patients are limited. Although clinically active in various solid cancers, these drugs are associated with typical adverse effects of anti-VEGF treatment, e.g., hypertension, thrombosis, emboli, bleeding, impaired wound healing, proteinuria, bowel perforation, and CNS disorders, which lead to treatment discontinuation in many patients [101]. Furthermore, many tumors are either inherently resistant or gradually develop adaptive resistance to VEGF pathway inhibition therapies [102]. This dictates the use of anti-VEGF treatments, like other targeted treatments, only according to selected molecular subgroups. 

In the GOG-86P trial (NCT00977574, [105]), researchers implemented translational research to examine results for common somatic mutations and microsatellite instability for associations with patients’ outcomes in each of three arms, containing either bevacizumab, temsirolimus, or ixabepilone, with standard paclitaxel and carboplatin in advanced or recurrent EC. Progression-free survival and overall survival were not increased in any of the experimental arms. However, in a post hoc analysis of the data, *CTNNB1* mutations (present in up to 20% of EC patients) were associated with a significantly increased PFS in bevacizumab-treated patients. *CTNNB1* mutations may be associated with increased VEGF expression and angiogenesis. They are primarily found in the subset of low-grade endometrioid tumors that have a higher risk of recurrence. The authors conclude that *CTNNB1* mutations may serve as a predictive biomarker for bevacizumab treatment.

Similarly, the assessment of *TP53* mutation status in the same trial showed that women with *TP53* mutant EC had both improved progression-free and overall survival when treated with bevacizumab and chemotherapy, whereas women with *TP53* wild-type tumors showed no difference in outcomes. This might be due to the cell cycle regulation disruption and enhanced angiogenesis in tumor cells due to the loss of wild-type p53 repression of pro-angiogenic factors including the bevacizumab target VEGF-A [118]. 

Another group of anti-angiogenic agents evaluated in clinical trials for use in EC patients are tyrosine kinase inhibitors of VEGFRs. Nintedanib showed modest activity with an objective response rate (ORR) of 9.4% in the treatment of advanced or recurrent EC and did not meet the study’s primary endpoint of efficacy. Nevertheless, preclinical trials on nintedanib suggest that it may be more effective in tumors with loss-of-function *TP53* mutations [112]. Monotherapy with cediranib for recurrent or persistent EC was well tolerated and showed sufficient activity (with a median PFS of 3.7 months and median OS of 12.5 months), which warranted further investigation for recurrent EC [111]. Brivanib was also well tolerated and worthy of further investigation as a single agent in recurrent or persistent EC, based on PFS at 6 months (30.2%). Rates of 6-month PFS were higher for endometrioid carcinoma (31.5%) or mixed epithelial subtypes (50%) compared with serous carcinoma (10%) [110]. The combination of levanitinib and pembrolizumab was assessed in the phase-II KEYNOTE 146 trial (NCT02501096, Table 1) in advanced EC patients. The EC cohort (n = 108) showed an ORR of 64% in the mismatch-repair-deficient group and 36% in the mismatch-repair-proficient group [116]. In July 2021 (after accelerated approval was granted in 2019), the FDA approved this combination for EC patients with advanced EC that is not microsatellite-instability-high or mismatch-repair-deficient and who exhibit disease progression following prior systemic therapy but are not candidates for curative surgery or radiation [119]. 

Aflibercept serves as a decoy receptor for VEGF binding at high affinity. In the Gynecologic Oncology Group Phase-II clinical trial, it met pretrial activity parameters but was associated with significant toxicity at the dose and schedule used [109]. Trebananib is an Fc fusion peptibody that prevents Tie2 receptor activation through binding of both Ang-1 and Ang-2. However, a phase-II trial showed an ORR of 3.1% for recurrent or persistent EC, with insufficient single-agent activity to warrant further investigation of trebananib [117].

## 7. Conclusions

Angiogenesis represents an important step in the pathogenesis of EC development, progression, and metastases and thus an opportunity for better diagnostic and tailored therapeutic approaches. There are conflicting results regarding the role of AFs in EC, and more clinical studies that evaluate circulating AFs are necessary to reach a uniform conclusion regarding the use of AFs as diagnostic and prognostic biomarkers. However, although the results from different studies regarding the use of AFs as biomarkers for gynecological cancer are not conclusive, there is a clear pattern of decreased progression-free survival and overall survival rates when pro-AFs are over-expressed in either serum or EC tissue. Relevant studies concluded that increased AF levels were correlated with worsening in the clinical stage and histological grade of EC and were associated with poorer prognosis.

Molecular changes occur earlier than phenotypic changes, and thus identifying new biomarkers may enable early diagnosis and facilitate decision making regarding the appropriate therapeutic (surgical and/or pharmacotherapeutic) measures. There is a trend toward a combined molecular and histological approach to risk stratification, and the discovery of robust prognostic biomarkers will eventually lead to improved survival outcomes for women with EC.

Anti-angiogenic therapy has already been incorporated into the regular treatment of several types of human cancers, including EC and other gynecological cancers. However, the anti-angiogenic agents in use today are only effective in a subset of patients, and many initial responders become resistant over time. This emphasizes the urgent need for a better understanding of the molecular and cellular effects of current anti-angiogenic agents as well as the discovery of alternative inhibitors of angiogenesis. As learned from recent clinical trials, proper design and conduct of translational research can yield important findings and allow assessment of treatment efficacy within biologically similar tumors. This may help to stratify patient populations for future treatment options.

## Figures and Tables

**Table 1:** Completed phase-II trials evaluating anti-angiogenic agents in recurrent/metastatic endometrial cancer. ORR—objective response rate; mPFS—median progression-free survival; mOS—median overall survival.

Trial ID	Agent	Anti-Angiogenic Target/ Mechanism of Action	N	ORR (%)	mPFS (Months)	mOS (Months)
NCT00025467 [97]	Thalidomide	VEGF	24	12	1.7	-
NCT00301964 [103]	Bevacizumab	VEGF	52	13.5	4.2	10.5
NCT00723255 [104]	Bevacizumab + temsirolimus	VEGF + combinations	49	24.5	5.6	16.9
NCT00977574 [105]	Bevacizumab + paclitaxel + carboplatin	VEGF + combinations	349	60.0	-	34.0
NCT01770171 [106]	Bevacizumab + paclitaxel + carboplatin	VEGF + combinations	108	74.4	13.7	40.0
NCT00879359 [107]	Bevacizumab + paclitaxel + carboplatin	VEGF + combinations	15	73	18	58
NCT01005329 [108]	Bevacizumab + cisplatin + radiotherapy	VEGF + combinations	34	-	79.1% at 2 years follow-up	96.7% at 2 years follow-up
NCT00462826 [109]	Aflibercept	Soluble VEGFR	44	6.8	2.9	14.6
NCT00888173 [110]	Brivanib	Tyrosine kinase inhibitor	43	18.6	3.3	10.7
NCT01132820 [111]	Cediranib	Tyrosine kinase inhibitor	48	12.5	3.7	12.5
NCT01225887 [112]	Nintedanib	Tyrosine kinase inhibitor	32	9.4	3.3	10.1
NCT00478426 [113]	Sunitinib	Tyrosine kinase inhibitor	33	18.1	3.0	19.4
NCT01111461 [114]	Lenvatinib	Tyrosine kinase inhibitor	133	14.3	5.4	10.6
NCT02501096 [115,116]	Lenvatinib + pembrolizumab	Tyrosine kinase inhibitor + immunotherapy	54	39.6	7.4	-
NCT01210222 [117]	Trebananib	Angiopoietins/Tie2	32	3.1	2.0	6.6

## Data Availability

Not applicable.
